# Associations of Butyrylcholinesterase Activity and Lipid-Related Indices with Obesity in Thai Children

**DOI:** 10.3390/ijerph22071107

**Published:** 2025-07-14

**Authors:** Kanjana Suriyaprom, Sujinda Songtrai, Nittiya Chowchaikong, Chutima Sirikulchayanonta

**Affiliations:** 1 Faculty of Medical Technology, Rangsit University, Paholyothin Road, Pathumthani 12000, Thailand; sujinda.s@rsu.ac.th (S.S.); nittiya.c@rsu.ac.th (N.C.); 2 Department of Preventive and Social Medicine, College of Medicine, Rangsit University, Bangkok 10400, Thailand; chutima.sir@rsu.ac.th

**Keywords:** butyrylcholinesterase, lipid-related indices, TyG-related indices, Thai children, obesity

## Abstract

Background: Childhood obesity is a significant global health concern. Butyrylcholinesterase (BChE) has been shown to play a role in lipid metabolism. This study aimed to assess BChE activity, obesity-related and lipid-related indices, and dyslipidemia in obese and non-obese children, and to investigate the associations of these parameters with obesity among Thai children. Methods: The study included 661 Thai children, consisting of 338 with obesity and 323 with a normal weight. Anthropometric measurements, metabolic parameters, obesity- and lipid-related indices, and BChE activity were evaluated. Results: The obese group exhibited significantly higher BChE activity and obesity-related and lipid-related indices compared to the non-obese group (*p* < 0.01). Additionally, metabolic parameters—including glucose levels, triglyceride-glucose (TyG) index, and TyG-related indices—as well as the lipid profile, which included triglycerides (TG), non-high-density lipoprotein cholesterol (non-HDL-C), and very-low-density lipoprotein cholesterol (VLDL-C), were all significantly elevated in the obese group (*p* < 0.01). Obesity was associated with dyslipidemia (*p* < 0.01). Moreover, BChE activity showed a positive correlation with obesity-related and lipid-related indices, along with several metabolic parameters (*p* < 0.002). The upper stratum of BChE activity (OR = 5.356), the non-HDL-C/HDL-C ratio (OR = 2.185), and the TG/HDL-C ratio (OR = 1.703) were found to be effective in evaluating and predicting the risk of obesity, even after adjusting for potential covariates (*p* < 0.01). Conclusions: These findings indicate a significant relationship between obesity and increased BChE activity, lipid-related indices, and dyslipidemia in Thai children. Therefore, changes in BChE activity may be considered a factor associated with obesity, enhancing its potential as a marker for obesity assessment.

## 1. Introduction

Obesity is a complex, multifactorial disease characterized by excessive fat accumulation. Childhood obesity is a pressing health issue worldwide, including in Thailand. Numerous studies indicate a strong relationship between childhood obesity and the persistence of obesity into adulthood, as well as the future development of non-communicable diseases such as hypertension, cardiovascular disease, and diabetes mellitus [1].

Butyrylcholinesterase (BChE), also known as pseudocholinesterase or serum cholinesterase, is a serine hydrolase related to acetylcholinesterase (AChE). It catalyzes the hydrolysis of acetylcholine and is involved in breaking down various substances, including succinylcholine, xenobiotics, and ghrelin—the hunger hormone associated with lipid metabolism [2,3]. BChE is primarily synthesized in the liver and is present in the nervous system and most human tissues, including adipose tissue. Notably, its concentration in plasma is higher than that of AChE [4]. Despite its significance, the precise function of BChE has not been fully understood. Previous studies have indicated a link between BChE function and lipid metabolism, particularly its role in lipid hydrolysis [3]. Animal experiments have shown that mice lacking BChE or *BChE* knockout (−/−) developed central obesity and exhibited an impaired lipid profile [5]. However, findings regarding BChE activity in humans and its relationship to obesity remain inconsistent. Some studies have reported increased BChE activity in obese individuals compared to those of normal weight [6,7], while others found no significant relationship [8] or suggested that lower BChE activity might be associated with increased fat accumulation and weight gain [9]. Although previous research has explored BChE activity, its physiological role is still unclear, especially concerning Thai children. There is limited information on BChE activity and its relationship to obesity and metabolic risk factors in this population. Therefore, the present study aims to assess BChE activity, obesity-related and lipid-related indices, and dyslipidemia in both obese and non-obese children, as well as to explore associations between these parameters and obesity among Thai children. To the best of our knowledge, this is the first study to evaluate these associations in Thai children.

## 2. Materials and Methods

### 2.1. Study Groups

This cross-sectional clinical study received approval from the Ethical Committee of the Research Institute of Rangsit University (RSUERB2020-035), Thailand, and adhered to the Declaration of Helsinki. The purposes of the study were carefully explained to both the children and their parents before obtaining written informed consent from the volunteer children or their parents. A total of 661 Thai children from primary schools in Bangkok and Pathum Thani, central Thailand, were selected during a health screening program conducted from December 2020 to January 2022. The study included 661 children aged 9 to 12 years, comprising 338 obese children (149 girls [44.1%] and 189 boys [55.9%]) and 323 non-obese children (173 girls [53.6%] and 150 boys [46.4%]). The sample size was calculated using a statistical power of 90% and a type I error of 0.05, based on a report by Boberg et al. [10] regarding the association between BChE levels and obesity (obese group: 6.5 ± 2.8 vs. non-obese group: 4.6 ± 1.1 for BChE levels). A single medical doctor conducted the medical history interviews and physical examinations throughout the study. Participants were excluded if they had significant medical conditions, such as lung, liver, kidney, gastrointestinal, cardiovascular, and infectious diseases. Additionally, those using prescription medications, vitamins, or dietary supplements were excluded from the study.

### 2.2. Anthropometric Measurements

Anthropometric measurements were obtained by a trained examiner using a calibrated digital weighing scale (Seca, Germany) and a microtoise (Seca^®^, Hamburg, Germany) to measure weight and height, respectively. Children, wearing light clothing, were instructed to stand on the scale without shoes and look straight ahead. Both weight and height were measured twice, with an accepted error of 0.1 kg and 0.5 cm, respectively. The Body Mass Index (BMI) was calculated and converted into BMI z-scores adjusted for age and gender according to the new World Health Organization reference [11]. The cut-off values for weight status were defined as follows: obese—BMI z-score > 2 SD, and normal weight—BMI z-score ≤ 1 SD to > −1 SD. Waist circumference (WC) was measured to the nearest 0.5 cm at the midpoint between the lower rib and the upper edge of the iliac crest using a flexible measuring tape. Blood pressure (BP) was measured on the left arm, with two readings taken 1 to 2 min apart after a 10 min rest in a seated position, conducted by a nurse.

### 2.3. Laboratory Measurements

Participants were required to fast for at least 12 h, preferably overnight, and 8 mL of venous blood was drawn in the morning. Levels of glucose, triglycerides (TG), total cholesterol (TC), high-density lipoprotein cholesterol (HDL-C), low-density lipoprotein cholesterol (LDL-C), alanine transaminase (ALT), and aspartate aminotransferase (AST) were measured using a Cobas c501 plus automatic biochemical analyzer with matched reagent kits (Roche Diagnostics GmbH, Mannheim, Germany). Additionally, the activity of heparinized plasma BChE was assessed using a Cobas c501 automatic analyzer with a cholinesterase reagent kit (04498577; Roche Diagnostics GmbH, Mannheim, Germany). The BChE assay kit is based on BChE’s ability to hydrolyze butyrylthiocholine into thiocholine and butyrate. Thiocholine reduces yellow hexacyanoferrate (III) to near colorless hexacyanoferrate (II), which can be measured photometrically. The intra- and inter-assay errors were approximately <5% and <8%, respectively.

### 2.4. Definition of Index

The Body Mass Index (BMI) was calculated as weight (kg) divided by height (m^2^). The waist-to-height ratio (WHtR) was computed by dividing the waist circumference (cm) by height (cm). The triglyceride and glucose index (TyG) was calculated using the equation: ln[fasting TG (mg/dL) × fasting plasma glucose (mg/dL)/2]. The TyG related to BMI (TyG-BMI), TyG related to WC (TyG-WC), and TyG related to WHtR (TyG-WHtR) were evaluated by multiplying the TyG index by BMI, WC, and WHtR, respectively [12]. The body shape index (ABSI) was defined as WC/(BMI^2^/3 × height^1^/2), expressing WC and height in meters [13]. Non-HDL-C was calculated as TC minus HDL-C. Very-low-density lipoprotein cholesterol (VLDL-C) was estimated by dividing TG by 5. Additionally, lipid indices were composed of ratios: TG to HDL-C (TG/HDL-C), TC to HDL-C (TC/HDL-C), LDL-C to HDL-C (LDL-C/HDL-C), and non-HDL-C to HDL-C (Non-HDL-C/HDL-C). The upper stratum of BChE activity was defined according to the cut-off value at the 75th percentile [6], which in this study was 11,000 U/L.

### 2.5. Statistical Analysis

Statistical analysis was performed using SPSS for Windows, version 11.5 (SPSS, Chicago, IL, USA). The normality of the sample distribution of each continuous variable was tested using the Kolmogorov–Smirnov test. In the present study, non-parametric tests were recommended when the data did not have a normal distribution. Quantitative data were expressed as median with interquartile range (25th–75th percentile), and the Mann–Whitney U-test was used for comparisons of parameters between the obese and non-obese groups. Spearman’s rho was used to search for relationships involving variables. To evaluate links between obesity as a dependent variable and other potential factors, binary logistic regression was applied. The goodness-of-fit of binary logistic regression models was established by the Hosmer–Lemeshow test. The level of statistical significance was set at *p* < 0.05.

## 3. Results

A total of 661 children aged 9 to 12 years were enrolled in this study. Table 1 provides a comparison of age, anthropometric–metabolic variables, and obesity- and lipid-related indices between the non-obese and obese groups. The age difference between the two groups was not statistically significant. However, BChE activity was significantly higher in the obese group compared to the non-obese group (*p* < 0.01). Additionally, various obesity-related indices, such as weight, WC, WHtR, ABSI, BMI, and BMI z-score, were significantly higher in the obese group (*p* < 0.01). Metabolic parameters, including systolic BP, diastolic BP, glucose, TyG index, TyG-related indices (TyG-BMI, TyG-WC, and TyG-WHtR), and lipid profile markers (TG, non-HDL-C, and VLDL-C) were also significantly elevated in the obese group. In contrast, the level of HDL-C was significantly lower in the obese group than in the non-obese group (*p* < 0.01). Furthermore, significant differences were found between the two groups in lipid-related indices, including LDL-C/HDL-C, TG/HDL-C, TC/HDL-C, and non-HDL-C/HDL-C ratios (*p* < 0.01). AST and ALT levels were significantly higher in the obese group compared to the non-obese group (*p* < 0.01).

Table 2 presents the frequency of specific types of dyslipidemia in both groups. There were significant differences between the obese group (55.6%) and the non-obese group (29.7%) regarding the frequency of dyslipidemia (*p* < 0.001). Furthermore, high TG, high LDL-C, and low HDL-C levels were significantly associated with obesity (*p* < 0.01), with increased LDL-C being the most common lipid abnormality, observed in 31.4% of obese children. In contrast, the frequency of high TC levels did not differ significantly between the two groups (*p* > 0.05).

The Spearman’s rank correlation test results are summarized in Table 3. To mitigate the risk of a Type I error or a false positive due to the calculation of numerous correlations (25 variables), the significance level of the correlation coefficients was adjusted using the Bonferroni correction. In the total group of children, BChE activity was negatively correlated with HDL-C (*p* < 0.002). Additionally, BChE activity was positively correlated with obesity-related indices, blood pressure, AST, ALT, the TyG index, TyG-related indices, and various lipid-related indices and profiles (*p* < 0.002), except for TC, LDL-C, and glucose. In the obese subgroup, BChE activity remained negatively correlated with HDL-C and positively correlated with obesity-related indices, markers of insulin resistance, liver enzymes, and some lipid-related indices (*p* < 0.002).

Logistic regression analysis was used when obesity was used as a dependent variable, and age, sex, the upper stratum of BChE activity, TyG index, TG/HDL-C ratio, and non-HDL-C/HDL-C ratio were taken as independent variables. Odds ratios (ORs) for potential associations between obesity and these variables are displayed in Table 4. Three variables were significantly associated with an increased risk of obesity: the upper stratum of BChE activity (OR = 5.356, *p* < 0.01), the non-HDL-C/HDL-C ratio (OR = 2.185, *p* < 0.01), and the TG/HDL-C ratio (OR = 1.703, *p* < 0.01). The Hosmer–Lemeshow goodness-of-fit test (χ^2^ = 14.13, *p* = 0.098) was not statistically significant, indicating that the model fit well with the data.

## 4. Discussion

To the best of our knowledge, the present study supports the association between BChE activity, lipid-related indices, and obesity in Thai children. Our results indicate that BChE activity is higher in obese children compared to their non-obese counterparts and is positively correlated with obesity and lipid-related indices. The physiological role of BChE remains unclear; however, previous research suggests it may help mitigate diet-induced weight gain by regulating energy expenditure, adipose tissue growth, and lipid metabolism in the liver [5]. Schopfer et al. also reported that BChE can regulate ghrelin’s physiological functions by cleaving n-octanoyl ghrelin, producing inactive des-acyl ghrelin, which may help decrease appetite [2]. Ghrelin, made in the stomach, stimulates appetite and promotes obesity [14]. Moreover, ghrelin is known to have anti-inflammatory effects by acting significantly on the innate and adaptive immune systems; it also regulates energy homeostasis by enhancing carbohydrate utilization while sparing fat, promoting lipogenesis and triglyceride uptake in white adipose tissue, and stimulating fatty acid oxidation in skeletal [15]. Evidence suggests that genetic deficiency in BChE results in ghrelin levels that are 50% above normal, whereas BChE-enhanced mice exhibit lower plasma ghrelin levels and resist obesity on a high-fat diet [5,16]. Previous studies show that individuals with low BChE activity are more prone to increased adiposity and weight gain [9,17]. In contrast, the study by Oliveira et al. found no significant difference in BChE activity between obese and non-obese women [8]. However, our findings support the notion that body size phenotypes and obesity-related indices—such as BMI, BMI z-score, WC, WHtR, and ABSI—are positively correlated with BChE activity, with obese children exhibiting higher BChE activity than those of normal weight. These results among Thai children are consistent with previous studies conducted on Chinese adolescents [6], Mexican children [7], and Brazilian adolescents [18]. Further research by Dantas et al. indicated that increasing BChE levels might be influenced by the ghrelin gene (GHRL), suggesting a consequential metabolic role in the complex lipid metabolism [19]. Thus, the rising levels of BChE in obesity may relate to the body’s adjustment of energy homeostasis by modulating the BChE–ghrelin axis. While the BChE–ghrelin axis appears to be a complex mechanism, the interconnected roles of ghrelin and BChE in the progression of obesity still warrant further investigation, particularly in children.

Additionally, Khanna et al. reported that elevated BChE activity could serve as a specific marker for detecting acute, chronic, low-grade systemic inflammation [20]. Acetylcholine, known to have anti-inflammatory properties, decreases in concentration as BChE activity increases [21]. Therefore, heightened BChE activity in obese children may reflect reduced acetylcholine levels and increased systemic inflammation. Excess adipose tissue can elevate the expression of inflammatory cytokines (such as TNF-α) and adipokines (like leptin and resistin), both of which contribute to the pathogenesis of insulin resistance by disrupting insulin signaling and action [22]. Therefore, obesity-induced chronic inflammation could be a key component linked with the pathogenesis of insulin resistance and the progression of metabolic disorders.

Our study also revealed that dyslipidemia is frequently observed in obese Thai children. Obesity, characterized by excessive fat deposits, poses a significant burden on current and future health, including increased mortality risk, with concerns such as non-communicable diseases [1]. The early onset of insulin resistance and dyslipidemia phenotypes are considered to be two major metabolic complications of childhood obesity, which can lead to future cardiovascular issues [23,24]. Prior research indicated that obese individuals are more likely to experience dyslipidemia, with varying prevalence rates reported worldwide. The prevalence of dyslipidemia in obese children has been reported to be as high as 62% in central Portugal [24], 38.23% in girls and 40.51% in boys in Poland [25], 56.7% in Turkey [26], and 55.3% in Abu Dhabi [27]. Our results indicated that the prevalence of dyslipidemia in the obese group of Thai children was 55.6%, consistent with findings from other populations and reinforcing the association between dyslipidemia and obesity. Moreover, recent studies have enhanced our understanding of the relationship between BChE activity and lipid metabolism, highlighting its potential role in maintaining lipid homeostasis, including lipolytic activity and the regulation of fatty acid use and storage [28]. Research confirming the lipolytic activity of BChE by Gok et al. demonstrated that purified human BChE functions as a lipase, efficiently hydrolyzing 4-mu palmitate and utilizing it as a substrate [3]. BChE expression and activity are influenced by circulating lipid levels and their storage in the liver and adipose tissue, as highlighted by Chen et al. [5]. An increase in the availability or flux of free fatty acids to the liver, along with heightened lipogenesis from carbohydrates, can lead to dyslipidemia and elevated BChE activity [28]. A study conducted on Mexican children showed a significant correlation between BChE activity, nutritional status, and TG levels [29]. Vallianou et al. also reported that BChE activity significantly correlated with various parameters of adiposity and lipid profiles [30]. Conversely, Stojanov et al. found no significant correlations between BChE activity and lipid profiles [31]. Our study involving Thai children aligns with previous findings [29,30], supporting the potential role of BChE in lipid metabolism. We observed negative correlations between BChE activity and HDL-C levels, as well as positive correlations between BChE activity and other lipid levels. However, information regarding the relationship between BChE activity and lipid-related indices (such as non-HDL-C/HDL-C, LDL-C/HDL-C, TG/HDL-C, and TC/HDL-C) and TyG-related indices (including TyG-BMI, TyG-WC, and TyG-WHtR) is scarce, particularly in children. These relationships are essential areas for further research. The TyG index serves as a reliable alternative biomarker for insulin resistance. Lim et al. reported that combining the TyG index with obesity indices—such as TyG-BMI, TyG-WC, and TyG-WHtR—was more effective than using the TyG index alone for detecting insulin resistance [32]. Although HOMA-IR (homeostasis model assessment of insulin resistance) is commonly employed to evaluate insulin resistance, it requires fasting glucose and insulin level measurements. However, measured insulin levels in blood are not routinely feasible in clinical practice. In contrast, the TyG index and its related indices are increasingly recognized as simple, convenient, reliable, and cost-effective surrogates for assessing insulin resistance [32]. Our study further revealed positive correlations between the TyG index, TyG-related indices, and lipid-related indices with BChE activity. To our knowledge, this is the first report to explore the association between BChE and TyG-related indices within a cohort of Thai children. Moreover, our findings support earlier studies conducted on German women [33] and Japanese adults [34], which identified a link between BChE levels and insulin resistance. Elevated BChE levels are likely associated with impaired glucose metabolism [33]. Research on the CaCo-2 intestinal cell line indicated that high insulin levels might stimulate BChE [35]. Heni et al. suggested that BChE activity could serve as a useful estimate for both insulin resistance and elevated liver fat content, due to BChE’s involvement in metabolic processes within hepatocytes that are crucial for glucose metabolism [33]. Furthermore, several epidemiological studies have indicated that proatherogenic lipid indices, such as TC/HDL-C, TG/HDL-C, non-HDL-C/HDL-C, and LDL-C/HDL-C, are superior to individual conventional lipid markers (such as TG, TC, HDL-C, and LDL-C) for predicting cardiovascular disease risk [36]. The non-HDL-C/HDL-C ratio may indicate combined lipid problems and is a better predictor of various dyslipidemia-related diseases [37]. The TG/HDL-C ratio also appears to be a better predictor of plasma atherogenicity and is closely associated with measures of adiposity, including BMI and body fat [38]. Our logistic regression analysis supported the conclusion that increased TG/HDL-C ratio, non-HDL-C/HDL-C ratio, and BChE activity are linked to a higher risk of obesity.

Nowadays, metabolic disturbances associated with obesity and non-alcoholic fatty liver disease (NAFLD) appear to begin at an early age in obese children. Our findings are consistent with a previous cross-sectional study that indicated elevated liver enzymes in prepubertal children with obesity when compared to their normal-weight counterparts [39]. NAFLD is often associated with increased levels of liver enzymes, and elevated ALT levels are a common indicator of liver inflammation or injury. However, Katoh et al. reported in a large cross-sectional study that serum cholinesterase is a better marker for fatty liver than ALT [40]. Our results also supported a positive association between liver enzymes (including ALT and AST) and BChE activity in Thai children. Increased BChE activity has frequently been observed in cases of NAFLD and can indicate higher fat infiltration in the liver [41]. Additionally, it may serve as a marker of low-grade systemic inflammation [20], which could be partially involved in the pathogenesis of NAFLD associated with plasma BChE levels [41]. Although the exact molecular mechanisms underlying the development and progression of NAFLD remain unclear, key aspects, including liver inflammation, increased oxidative stress, and fat accumulation—all linked to insulin resistance —are significant factors that connect BChE activity to liver enzymes, particularly in obesity [33,42].

It is important to acknowledge certain limitations in our study. First, we did not assess ghrelin and insulin levels, nor did we account for external factors such as diet and physical activity. Additionally, we did not directly evaluate the distribution of body fat and its visceral deposits using computed tomography. Finally, our data are cross-sectional, so we have not been able to ascertain causality in associations. Therefore, future longitudinal studies should focus on validating population-specific effect sizes, particularly in children, and probe the causal links between the BChE–ghrelin axis and obesity progression, its metabolic consequences, and external factors in large children’s populations.

## 5. Conclusions

The current study involving Thai children suggests a significant relationship between obesity and increased BChE activity, lipid-related indices, and dyslipidemia. Furthermore, BChE activity shows a positive correlation with lipid levels and indices related to obesity and insulin resistance. These findings imply that the rise in BChE activity associated with obesity may serve as a compensatory mechanism in response to metabolic impairments related to lipid metabolism or an attempt to restore energy balance. Additionally, changes in BChE activity may be regarded as one of the obesity-related factors for improving the potential of obesity assessment. Understanding the mechanisms behind these changes in the early stages of obesity is crucial for recognizing and preventing the risk of developing obesity-related non-communicable diseases in the future.

## Figures and Tables

**Table 1:** Comparison of the age, anthropometric–metabolic variables, and obesity- and lipid-related indices between non-obese and obese children.

Variables	Obese Group (*n* = 338)	Non-Obese Group (*n* = 323)	*p*-Value
Age (years)	11.0 (9.0–12.0)	11.0 (9.0–12.0)	0.751
Weight (kg)	54.5 (39.0–87.4)	35.5 (24.2, 48.0)	<0.001 **
Height (cm)	147.5 (132.0–164.5)	144.5 (125.5, 161.5)	<0.001 **
WC (cm)	82.5 (66.5, 104.5)	61.0 (51.0, 70.5)	<0.001 **
WHtR	0.58 (0.46, 0.68)	0.42 (0.36, 0.50)	<0.001 **
ABSI (m^11/6^/kg^2/3^)	0.0803 (0.0768, 0.0830)	0.0761 (0.0736, 0.0791)	<0.001 **
BMI (kg/m^2^)	25.5 (22.0, 33.2)	17.6 (16.5, 21.0)	<0.001 **
BMI z-score	2.6 (2.1, 4.2)	−0.1(−0.9, 0.9)	<0.001 **
Systolic BP (mmHg)	110.0 (85.5, 130.0)	98.0 (77.0, 120.0)	<0.001 **
Diastolic BP (mmHg)	70.0 (56.0, 92.0)	65.0 (50.0–85.0)	<0.001 **
Glucose (mg/dL)	91.0 (81.0, 101.0)	89.0 (75.0, 100.0)	<0.001 **
TyG index	8.2 (7.5, 9.0)	7.9 (7.3, 8.5)	<0.001 **
TyG-BMI	207.5 (167.5, 290.0)	135.0 (114.5, 171.0)	<0.001 **
TyG-WC	681.1 (621.8, 747.0)	481.3 (445.5, 520.6)	<0.001 **
TyG-WHtR	4.7 (4.3, 5.0)	3.4 (3.2, 3.6)	<0.001 **
TC (mg/dL)	181.0 (131.0, 235.0)	185.0 (132.0, 225.0)	0.180
TG (mg/dL)	81.0 (46.0, 178.0)	60.0 (40.0, 140.0)	<0.001 **
LDL-C (mg/dL)	117.0 (67.0, 170.0)	114.0 (69.0, 158.0)	0.252
HDL-C (mg/dL)	46.0 (35.0, 67.0)	56.0 (39.0, 79.0)	<0.001 **
Non-HDL-C (mg/dL)	134.0 (85.0, 190.0)	128.0 (84.0, 170.0)	0.002 **
VLDL-C (mg/dL)	16.0 (8.0, 36.0)	12.0 (7.0, 27.0)	<0.001 **
LDL-C/HDL-C	2.5 (1.3, 4.0)	2.0 (1.1, 3.1)	<0.001 **
TG/HDL-C	1.7 (0.8, 5.1)	1.1 (0.6, 3.0)	<0.001 **
TC/HDL-C	3.9 (2.5, 6.0)	3.2 (2.3, 4.6)	<0.001 **
Non-HDL-C/HDL-C	2.9 (1.5, 5.0)	2.3 (1.3, 3.5)	<0.001 **
AST (IU/L)	24.0 (16.0, 41.0)	23.0 (15.0, 34.0)	0.009 **
ALT (IU/L)	21.0 (8.0, 40.0)	12.0 (7.0, 27.0)	<0.001 **
BChE (IU/L)	10,749 (7027, 14,005.0)	9106 (6711.0, 12,365.0)	<0.001 **

Note: All data are median (interquartile range). *p*-values are given for comparisons between groups tested with the Mann–Whitney U-test. Significance levels: ** *p* < 0.01. Abbreviations: ABSI, a body shape index; ALT, alanine aminotransferase; AST, aspartate aminotransferase; BChE, butyrylcholinesterase; BMI, body mass index; HDL-C, high-density lipoprotein-cholesterol; LDL-C, low-density lipoprotein-cholesterol; TG, triglycerides; TyG, triglycerides and glucose index; TC, total cholesterol; VLDL-C, very-low-density lipoprotein cholesterol; WHtR, waist-to-height ratio; WC, waist circumference.

**Table 2:** Frequencies of high TG, high TC, high LDL-C, low HDL-C levels, and dyslipidemia among non-obese and obese children.

Lipid Levels	Obese *n* (%)	Non-Obese *n* (%)	*p*-Value
TG levels			
≥130 mg/dL	48 (14.2%)	13 (4.0%)	<0.001 **
<130 mg/dL	290 (85.8%)	310 (96.0%)	
TC levels			
≥200 mg/dL	97 (28.7%)	74 (22.9%)	0.092
<200 mg/dL	241 (71.3%)	249 (77.1%)	
LDL-C levels			
≥130 mg/dL	106 (31.4%)	68 (21.1%)	0.002 **
<130 mg/dL	232 (68.6%)	255 (78.9%)	
HDL-C levels			
<40 mg/dL	72 (21.3%)	15 (4.6%)	<0.001 **
≥40 mg/dL	266 (78.7%)	308 (95.4%)	
Dyslipidemia			
Yes	188 (55.6%)	96 (29.7%)	<0.001 **
No	150 (44.4%)	227 (70.3%)	

Note: *p*-values are given for the results of the chi-square test. Significance levels: ** *p* < 0.01. Abbreviations: HDL-C, high-density lipoprotein-cholesterol; LDL-C, low-density lipoprotein-cholesterol; TC, total cholesterol; TG, triglycerides.

**Table 3:** Correlation coefficients of BChE concentration with other parameters.

Variables	BChE		
	Total Children	Obese Children	Non-Obese Children
Weight	0.375 **	0.127	0.098
WC	0.480 **	0.206 **	0.087
WHtR	0.527 **	0.254 **	0.231 **
ABSI	0.359 **	0.243 **	0.201 **
BMI	0.482 **	0.187 **	0.086
BMI z-score	0.519 **	0.230 **	0.170
Systolic BP	0.300 **	0.132	0.101
Diastolic BP	0.260 **	0.153	0.095
Glucose	0.045	0.057	0.010
TyG index	0.360 **	0.215 **	0.225 **
TyG-BMI	0.509 **	0.190 **	0.150
TyG-WC	0.519 **	0.254 **	0.159
TyG-WHtR	0.555 **	0.299 **	0.327 **
TC	0.085	0.150	0.111
TG	0.360 **	0.214 **	0.227 **
LDL-C	0.105	0.120	0.112
HDL-C	−0.220 **	−0.170 **	−0.097
Non-HDL-C	0.181 **	0.176 **	0.118
VLDL-C	0.361 **	0.224 **	0.228 **
LDL-C/HDL-C	0.239 **	0.135	0.097
TG/HDL-C	0.370 **	0.198 **	0.145
TC/HDL-C	0.285 **	0.116	0.101
Non-HDL-C/HDL-C	0.295 **	0.191 **	0.095
AST	0.230 **	0.208 **	0.195 **
ALT	0.401 **	0.213 **	0.101

Note: Spearman’s rank correlation test was performed. Bonferroni correction was used to adjust *p*-values and statistical significance: ** *p*-values < 0.002. Abbreviations: ABSI, a body shape index; ALT, alanine aminotransferase; AST, aspartate aminotransferase; BChE, butyrylcholinesterase; BMI, body mass index; HDL-C, high-density lipoprotein-cholesterol; LDL-C, low-density lipoprotein-cholesterol; TG, triglycerides; TC, total cholesterol; TyG, triglycerides and glucose index; VLDL-C, very-low-density lipoprotein cholesterol; WHtR, waist-to-height ratio; WC, waist circumference.

**Table 4:** Logistic regression and odds ratios (ORs) for potential associations between obesity and the upper stratum of BChE activity, the TyG index, TG/HDL-C ratio, and non-HDL-C/HDL-C ratio, with adjustments for potential confounding factors.

Variables	β	Odds Ratios	95% CI	*p*-Value
The upper stratum of BChE activity	1.678	5.356	3.358–8.543	<0.001 **
TyG index	0.438	1.549	0.581–3.130	>0.05
TG/HDL-C	0.532	1.703	1.284–2.258	<0.001 **
non-HDL-C/HDL-C	0.782	2.185	1.596–2.258	<0.001 **

Note: Multiple logistic regression analyses were performed, and ORs (95% CIs) are shown with adjustments for age and gender. The 95% CI is the 95% confidence interval of the odds ratio. Significance levels: ** *p* < 0.01. Abbreviations: BChE, butyrylcholinesterase; HDL-C, high-density lipoprotein-cholesterol; TG, triglycerides; TyG, triglycerides and glucose index.

## Data Availability

The original contributions presented in this study are included in the article. Further inquiries can be directed to the corresponding author.

## Abbreviations

The following abbreviations are used in this manuscript:

ABSI	A body shape index
AChE	Acetylcholinesterase
AST	Aspartate aminotransferase
ALT	Alanine transaminase
BChE	Butyrylcholinesterase
BMI	Body mass index
BP	Blood pressure
CI	Confidence interval
HDL-C	High-density lipoprotein cholesterol
LDL-C	Low-density lipoprotein cholesterol
non-HDL-C	Non-high-density lipoprotein cholesterol
TC	Total cholesterol
TG	Triglycerides
TyG	Triglyceride-glucose index
VLDL-C	Very-low-density lipoprotein cholesterol
WC	Waist circumference
WHtR	Waist-to-Height ratio
